# Better outcome after pediatric resuscitation is still a dilemma

**DOI:** 10.4103/0974-2700.66524

**Published:** 2010

**Authors:** Sandeep Sahu, Kamal Kishore, Indu Lata

**Affiliations:** 1 Department of Anaesthesiology, Sanjay Gandhi Post Graduate Institute of Medical Sciences, Lucknow, UP, India; 2 Department of Anaesthesiology, Sanjay Gandhi Post Graduate Institute of Medical Sciences, Lucknow, UP, India; 3 Maternal & Reproductive Health, Sanjay Gandhi Post Graduate Institute of Medical Sciences, Lucknow, UP, India

**Keywords:** CPR, guidelines, outcome, out-of-hospital cardiac arrest, in-hospital cardiac arrest, pediatric resuscitation

## Abstract

Pediatric cardiac arrest is not a single problem. Although most episodes of pediatric cardiac arrest occur as complications and progression of respiratory failure and shock. Sudden cardiac arrest may result from abrupt and unexpected arrhythmias. With a better-tailored therapy, we can optimize the outcome. In the hospital, cardiac arrest often develops as a progression of respiratory failure and shock. Typically half or more of pediatric victims of in-hospital arrest have pre-existing respiratory failure and one-third or more have shock, although these figures vary somewhat among reporting hospitals. When in-hospital respiratory arrest or failure is treated before the development of cardiac arrest, survival ranges from 60% to 97%. Bradyarrthmia, asystole or pulseless electric activity (PEA) were recorded as an initial rhythm in half or more of the recent reports of in-hospital cardiac arrest, with survival to hospital discharge ranging from 22% to 40%. Data allowing characterization of out of hospital pediatric arrest are limited, although existing data support the long-held belief that as with hospitalized children, cardiac arrest most often occurs as a progression of respiratory failure or shock to cardiac arrest with bradyasystole rhythm. Although VF (Ventricular fibrillation, is a very rapid, uncoordinated, ineffective series of contractions throughout the lower chambers of the heart. Unless stopped, these chaotic impulses are fatal) and VT (Ventricular tachycardia is a rapid heartbeat that originates in one of the ventricles of the heart. To be classified as tachycardia, the heart rate is usually at least 100 beats per minute) are not common out-of-cardiac arrest in children, they are more likely to be present with sudden, witnessed collapse, particularly among adolescents. Pre-hospital care till the late 1980s was mainly concerned with adult care, and the initial focus for pediatric resuscitation was provision of oxygen and ventilation, with initial rhythm at the time of emergency medical services arrival being infrequently recorded. In the 1987 series, pre-hospital pediatric cardiac arrest demonstrated asystole in 80%, PEA in 10.5% and VF or VT in 9.6%. Only 29% arrests were witnessed, however, and death in many victims was caused by sudden infant death syndrome.

## INTRODUCTION

Cardiopulmonary resuscitation (CPR) is often performed poorly in children. High-quality CPR is especially important because effective pediatric advanced life support (ALS) depends on good pediatric basic life support (BLS). In the 21^st^ century, most children having in-hospital cardiac arrest attain return of spontaneous circulation and more than 25% survive to discharge,[1] but only approximately 10% survive to discharge following an out-of-hospital cardiac arrest (OHCA).[2] These survival rates indicate that resuscitation efforts are not futile. Because CPR is often performed poorly in both in-hospital and out-of-hospital settings, it is reasonable to assume that outcomes can be improved by a greater emphasis on providing prompt, good-quality CPR.[3,4]

However, children are different. Appropriate pediatric CPR differs from the CPR in adults because children are anatomically and physiologically different from adults. In addition, the pathogenesis of cardiac arrest and the most common rhythm disturbances are different in children. In contrast to adults, children rarely suffer from sudden ventricular fibrillation cardiac arrest from coronary artery diseases. Paediatric arrest, usually secondary to profound hypoxia or asphyxia, is caused by respiratory failure or circulatory shock. By the time the arrest occurs, all organs of the body have generally suffered significant hypoxia ischemic insult. The use of closed cardiac massage to provide adequate circulation during cardiac arrest was initially demonstrated in small dogs that had compliant chest walls.[5] Based on reasonable extrapolation, the investigation felt that closed-chest massage would be effective with children, but might not have a similar effect with adults. Therefore, the first patients successfully treated with closed-chest massage were children. Later investigations indicated that blood could also be circulated during CPR by the thoracic pump mechanism.[6] Regardless of the mechanism, cardiac output during CPR seems to be greater in children who have compliant chest walls. Recently, the American Heart Association (AHA) national registry CPR data indicated that outcome from in-hospital cardiac arrest is substantially better in infants than in older children, perhaps because of the superior perfusion during CPR.

## REVIEW OF THE LITERATURE

Recent reviews on CPR indicate that pediatric OHCA is an uncommon event and is generally described as having exceptionally poor survival with severe neurological sequalae. Population-based data for pediatric cardiac arrest are scant and are largely from urban areas. Some investigators have questioned whether resuscitation of children with OHCA is warranted because of the reported poor outcomes and associated high financial cost. The Resuscitation Outcomes Consortium (ROC) Epistry–Cardiac Arrest of 2009 is a population-based emergency medical services (EMS) registry of OHCA. This study examined the age-stratified incidence and outcomes of OHCA for 621 persons <20 years of age, who received CPR or defibrillation by EMS providers and/or received bystander automatic external defibrillator shock or who were pulseless but received no resuscitation by EMS between December 2005 and March 2007. Incidences of pediatric OHCA was 8.04/100,000 person/years, but 10-fold greater for infants <1 year (72.71%) of age, approaching the incidence of OHCA in adults. Survival for all pediatric OHCA was 6.4% versus 4.5% for adults. Pediatric patients were more likely to survive to discharge than adults (6.4% versus 4.5%). When adjusted for age group, witnessed arrest, obvious cause of arrest, sex and site, only age group and witnessed arrest were statistically associated with survival. Survival to discharge was more common among children and adolescents than among infants or adults. This study demonstrates that commonly reported overall survival figures are heavily influenced by very poor infant survival, whereas children and adolescents have substantially greater survival compared with adults.[8]

Review of the records of 101 children (median age, 2 years) with apnoea or no palpable pulse (or both) who presented to the Hospital for Sick Children in Toronto is a good example. Overall, there was a return of vital signs in 64 of the 101 patients; 15 survived to discharge from the hospital and 13 were alive 12 months after discharge. Factors that predicted survival to hospital discharge included a short interval between the arrest and arrival at the hospital, a palpable pulse on presentation, a short duration of resuscitation in the emergency department(ED) and the administration of fewer doses of epinephrine. No patients who required more than two doses of epinephrine or resuscitation for longer than 20 min in the emergency department survived to hospital discharge. The survivors who were neurologically normal after arrest had respiratory arrest only and were resuscitated within 5 min after arrival in the emergency department. Out of the 80 patients who had had a cardiac arrest, only six survived to hospital discharge, and all had neurologic sequalae. These results suggest that OHCA among children has a very poor prognosis, especially when efforts at resuscitation continue for longer than 20 min and require more than two doses of epinephrine.[9]

Patients with an initial rhythm of VT/VF have better survival than those with asystole/pulseless electric activity because of the reported poor outcomes and associated high financial cost.[10] In another study of 2009, pediatric cardiac arrest has traditionally been considered a futile medical condition with dismal outcomes. Data in the 21^st^ century indicate that more than 25% of the children treated for in-hospital cardiac arrests survive to hospital discharge and that more than 10% of the children older than 1 year treated for OHCAs survive to hospital discharge. Before arrest, exciting new studies demonstrate that the implementation of in-hospital pediatric medical emergency teams is associated with significant decreases in the incidence of cardiac arrest and overall pediatric hospital mortality. During arrest, ventricular fibrillation or ventricular tachycardia, once thought to be rare in children, occurs during 25% of in-hospital pediatric cardiac arrests and at least 7% of out-of-hospital pediatric cardiac arrests. Survival to hospital discharge is much more likely after arrests with a first-documented rhythm of VF or VT than after pulseless electric activity (PEA) and asystole. However, ventricular fibrillation or ventricular tachycardia is not always a favourable rhythm as survival to discharge is much less likely when ventricular fibrillation or ventricular tachycardia occurs during resuscitation from an arrest with the first documented rhythm of PEA or asystole. Further, extracorporeal membrane oxygenation (ECMO) CPR appears promising under special resuscitation circumstances to improve outcome from highly selected in-hospital pediatric cardiac arrest victims. Further, post-resuscitation interventions such as goal-directed therapies and therapeutic hypothermia have been demonstrated in adults and infants to improve the outcome for selected cardiac arrest victims, and are promising candidate targets for study in children. Pediatric cardiac arrest is not a futile condition; many children are successfully resuscitated each year. The implementation of new pre-arrest, intraarrest and post-resuscitative therapies has the potential to further improve the survival rates following pediatric cardiac arrest.[11]

Exciting discoveries in 2008 in basic and applied-science laboratories are now relevant for specific subpopulations of pediatric cardiac arrest victims and circumstances (e.g., ventricular fibrillation, neonates, congenital heart disease, ECMO). Improving the quality of interventions is increasingly recognized as a key factor for improving outcomes. Evolving training strategies include simulation training, just in time, just-in-place training, and crisis-team training. The difficult issue of when to discontinue resuscitative efforts is addressed. Outcomes from pediatric cardiac arrests are improving. Advances in resuscitation science and state-of-the-art implementation techniques provide the opportunity for further improvement in outcomes among children after cardiac arrest.[12]

Identifying patients in the out-of-hospital setting who have no realistic hope of surviving an OHCA could enhance utilization of scarce health care resources. To validate out-of-hospital termination-of-resuscitation two rules were developed by the Ontario Prehospital Life Support (OPALS) study group. One rule is for use by responders who are providing BLS and another for those providing the ALS. It was a retrospective cohort study using surveillance data prospectively submitted by emergency medical systems and hospitals in eight US cities to the Cardiac Arrest Registry to Enhance Survival (CARES) between October 2005 and April 2008. Case patients were 7235 adults with OHCA; of these, 5505 met the inclusion criteria. The overall rate of survival to hospital discharge was 7.1% (*n* = 392). Of the 2592 patients (47.1%) who met the BLS criteria for termination of resuscitation efforts, only five (0.2%) patients survived to hospital discharge. Of the 1192 patients (21.7%) who met the ALS criteria, none survived to hospital discharge. In this validation study, the BLS and ALS termination-of-resuscitation rules performed well in identifying patients with OHCA who had little or no chance of survival.[13]

The health and policy implications of regional variation in incidence and outcome of OHCA remain to be determined. Prospective observational studies of all OHCAs in done in ten North American sites (eight US and two Canadian) from May 2006 to April 2007 were followed-up to hospital discharge. Among the 10 sites, the total catchment population was 21.4 million, and there were 20,520 cardiac arrests. A total of 11,898 (58.0%) had resuscitation attempted; 2729 (22.9% of those treated) had initial rhythm of ventricular fibrillation or ventricular tachycardia or rhythms that were shockable by an automated external defibrillator 954 (4.6% of the total) were discharged alive. In this study involving 10 geographic regions in North America, there were significant and important regional differences in the OHCA incidence and outcome.[14]

## PEDIATRIC BLS AND ALS AS PER THE AHA, 2005

The following is a summary of the most important changes in recommendations for pediatric resuscitation since the last ILCOR review in 2000.[15,16] The scientific evidence supporting these recommendations is summarized in this document in AHA in collaboration with International Liaison Committee on Resuscitation. Guidelines 2005 for CPR and Emergency Cardiovascular Care: International Consensus on Science.[7]

1. Emphasis on the quality of CPR is increased: “Push hard, push fast, minimize interruptions; allow full chest recoil, and don’t hyperventilate”
  - Recommended chest compression-ventilation ratio:
2. For 1 lay rescuer and lone healthcare provider: 30:2
3. For healthcare providers performing 2-rescuer CPR: 15:2
  - Either the 2- or 1-hand technique is acceptable for chest compressions in children
  - 1 initial shock followed by immediate CPR for attempted defibrillation, instead of 3 stacked shocks
4. Biphasic attenuated shocks with an automated external defibrillator (AED) are acceptable for children ≥1 year of age
5. Routine use of high-dose epinephrine is no longer recommended
6. Either cuffed or uncuffed tracheal tubes are acceptable in infants and children
7. Exhaled CO2 monitoring is recommended for confirmation of tracheal tube placement and during transport
8. Consider induced hypothermia for patients who remain comatose following resuscitation
9. Emphasis is increased on intravascular (intravenous [IV] and intraosseous [IO]) rather than tracheal administration of drugs

## INITIAL STEPS OF CPR

The ILCOR Pediatric Task Force reviewed 45 topics related to pediatric resuscitation and treatment recommendation published in Circulation (2005; 112: III-73-III-90), which were the following:

Earlier CPR sequence differences were based on arrest etiology according to age. Resuscitation results might be improved if the sequence of lay rescuer CPR actions (i.e., the priority of phoning for professional help, getting an AED and providing CPR) is based on the etiology of cardiac arrest rather than age.

One of the most challenging topics debated during the 2005 Consensus Conference was the compression-ventilation ratio. The only data addressing a compression-ventilation ratio greater than 15:2 came from mathematical models. The experts acknowledged the educational benefit of simplifying training for lay rescuers (specifically 1-rescuer CPR) by adopting a single ratio for infants, children and adults with the hope that simplification might increase the number of bystanders who will learn, remember and perform CPR. On this basis, experts agreed that this single compression-ventilation ratio should be 30:2. Health care providers will learn the 2-person CPR and, for them, the recommended compression-ventilation ratio for two rescuers is 15:2.

### Activating EMS and getting the AED

A period of immediate CPR before phoning EMS and getting the AED (call fast) is indicated for most pediatric arrests because they are presumed to be asphyxial or prolonged.[17–20] In a witnessed sudden collapse (e.g., during an athletic event), the cause is more likely to be VF, and the lone rescuer should phone for professional help and get the AED (when available) before starting CPR and using the AED, if appropriate. Rescuers should perform CPR with minimal interruptions in chest compressions until attempted defibrillation.[21,22] In summary, the priorities for unwitnessed or non-sudden collapse in children are as follows: start CPR immediately/activate EMS > get the AED.[24–27]

While priorities for witnessed sudden collapse in children are as follows: activate EMS/get the AED > start CPR > attempt defibrillation.[28–33]

### Pulse check

Lay rescuers should start chest compressions for an unresponsive infant or child who is not moving or breathing.[34,36,39] Healthcare professionals may also check for a pulse but should proceed with CPR if they cannot feel a pulse within 10 sec or are uncertain whether a pulse is present.[35–40]

### Ventilations in infants

There are no data to justify a change from the recommendation that the rescuer attempts mouth-to-mouth and mouth-to-nose ventilation for infants. Rescuers who have difficulty achieving a tight seal over the mouth and nose of an infant, however, may attempt either mouth-to-mouth or mouth-to-nose ventilation.[35]

### Circumferential versus 2-finger chest compressions

The two-thumb-encircling hands chest compression technique with thoracic squeeze is the preferred technique for 2-rescuer infant CPR.[41–43] The 2-finger technique is recommended for 1-rescuer infant CPR to facilitate rapid transition between compression and ventilation and to minimize interruptions in chest compressions. It remains an acceptable alternative method of chest compressions for 2 rescuers.

### One- versus 2-hand chest compression technique

Both the 1- and 2-hand techniques for chest compressions in children are acceptable provided that rescuers compress over the lower part of the sternum to a depth of approximately one-third the anterior–posterior diameter of the chest.[45] To simplify education, rescuers can be taught the same technique (i.e., 2-hand) for adult and child compressions.

### Compression–ventilation ratio

For ease of teaching and retention, a universal compression–ventilation ratio of 30:2 is recommended for the lone rescuer responding to infants (for neonates, 3:1 ratio), children and adults. For healthcare providers performing 2-rescuer CPR, a compression–ventilation ratio of 15:2 is recommended. When an advanced airway is established (e.g., a tracheal tube, esophageal–tracheal combitube [Combitube] or laryngeal mask airway [LMA]), ventilations are given without interrupting chest compressions.

### Some CPR versus no CPR

Bystander CPR is important for survival from cardiac arrest. Trained rescuers should be encouraged to provide both ventilations and chest compressions. If rescuers are reluctant to provide rescue breaths, however, they should be encouraged to perform chest compressions alone without interruption.[46,47]

### Disturbances in cardiac rhythm

Evidence evaluation for the treatment of hemodynamically stable arrhythmias focused on vagal manoeuvres, amiodarone and procainamide. There were no new data to suggest a change in the indications for vagal manoeuvres or procainamide. Several case series described the safe and effective use of amiodarone in children, but these studies involved selected patient populations (often with post-operative arrhythmias) treated by experienced providers in controlled settings. Although there is no change in the recommendation for amiodarone as a treatment option in children with stable arrhythmias, providers are encouraged to consult with an expert knowledgeable in pediatric arrhythmias before initiating drug therapy.

There is insufficient evidence to identify an optimal shock waveform, energy dose and shock strategy (e.g., fixed versus escalating shocks, one versus three stacked shocks) for defibrillation. The new recommendation for the sequence of defibrillation in children is based on extrapolated data from adult and animal studies with biphasic devices, data documenting the high rates of success for first shock conversion of VF with biphasic waveforms and knowledge that interruption of chest compressions reduces coronary perfusion pressure. Thus, a 1-shock strategy may be preferable to the 3-shock sequence recommended in the ECC Guidelines 2000.[16] Many, but not all, AED algorithms have been shown to be sensitive and specific for recognizing shockable arrhythmias in children. A standard AED (“adult” AED with adult pad-cable system) can be used for children older than about 8 years of age and weighing more than about 25 kg. Many manufacturers now provide a method for attenuating the energy delivered to make the AED suitable for smaller children (e.g., use of a pad-cable system or an AED with a key or switch to select a smaller dose).

### Pediatric defibrillation

The treatment of choice for pediatric VF/pulseless VT is prompt defibrillation, although the optimum dose is unknown.[48–50] For manual defibrillation, we recommend an initial dose of 2 J/kg (biphasic or monophasic waveform).[51] If this dose does not terminate VF, subsequent doses should be 4 J/kg.[52–54]

For automated defibrillation (AED), it is recommended that the AED have both a high specificity in recognizing pediatric shockable rhythms and a pediatric dose-attenuating system to reduce the dose delivered by the device. We recommend an initial pediatric attenuated dose for children 1–8 years of age and up to about 25 kg (55 pounds) and 127 cm (50 inches) in length. There is insufficient information to recommend for or against the use of an AED in infants <1 year of age. A variable dose manual defibrillator or an AED able to recognize pediatric shockable rhythms and equipped with dose attenuation is preferred. In an emergency if such a defibrillator is not available, a standard AED with standard electrode pads may be used. A standard AED (without a dose attenuator) should be used for children ≥25 kg (about 8 years of age) and older adolescent and adult victims.[55,56] Turn the AED on, follow the AED prompts, and resume chest compressions immediately after the shock. Minimize interruptions in chest compressions.

### Management of shock-resistant VF/pulseless VT

IV amiodarone can be considered as part of the treatment of shock-refractory or recurrent VT/VF.[55]

### Airway and ventilation

Maintaining a patent airway and ventilation are fundamental to resuscitation. Adult and animal studies during CPR suggest detrimental effects of hyperventilation and interruption of chest compressions. For children requiring airway control or ventilation for short periods in the out-of-hospital setting, bag-valve–mask ventilation produces equivalent survival rates compared with ventilation with tracheal intubation.[57,58] The risks of tracheal tube misplacement, displacement and obstruction are well recognized and an evidence-based review led to a recommendation that proper tube placement and patency be monitored by exhaled CO_2_ throughout transport. A review also found that cuffed tracheal tubes could be safely used in infants also.

Following the return of spontaneous circulation from cardiac arrest, toxic oxygen by-products (reactive oxygen species, free radicals) are produced that may damage the cell membranes, proteins and DNA (reperfusion injury). There are no clinical studies in children outside the newborn period comparing different concentrations of inspired oxygen during and immediately after resuscitation, and it is therefore difficult to differentiate “sufficient” from “excessive.”

### Advanced airways

Advanced airways include the tracheal tube, the Combitube and the LMA. Experts at the 2005 Consensus Conference reviewed the available evidence on use of the tracheal tube and LMA in infants and children.[59] There were no data on the use of the Combitube in this age group.

### Oxygen

There is insufficient information to recommend for or against the use of any specific inspired oxygen concentration during and immediately after resuscitation from cardiac arrest. Until additional evidence is published, we support healthcare providers’ use of 100% oxygen during resuscitation (when available).[60] Once circulation is restored, providers should monitor oxygen saturation and wean inspired oxygen while ensuring adequate oxygen delivery.[61]

### Vascular access and drugs for cardiac arrest

Vascular access can be difficult to establish during resuscitation of children. Review of the evidence showed increasing experience with IO access and resulted in a de-emphasis of the tracheal route for drug delivery.[62,63] Evidence evaluation of resuscitation drugs was limited by a lack of reported experience in children. There was little experience with vasopressin in children in cardiac arrest and inconsistent results in adult patients. In contrast, there was a good study in children showing no benefit, and possibly some harm, in using high-dose epinephrine for cardiac arrest.

### Post-resuscitation care

Post-resuscitation care is critical to a favourable outcome. An evidence-based literature review was performed on the topics of brain preservation and myocardial function after resuscitation from cardiac arrest. It showed the potential benefits of induced hypothermia on brain preservation, the importance of preventing or aggressively treating hyperthermia, the importance of glucose control and the role of vasoactive drugs in supporting hemodynamic function.

### Predictors of outcome in children

Multiple studies in adults have linked characteristics of the patient or of the cardiac arrest with prognosis following in-hospital or OHCA. Experience in children is more limited. Six pediatric studies[64–69] show that prolonged resuscitation is associated with a poor outcome. Although the likelihood of a good outcome is greater with a short duration of CPR, two pediatric studies reported good outcomes in some patients following 30–60 min of CPR in the in-patient setting when the arrests were witnessed and prompt and presumably excellent CPR was provided.[70,71] Children with cardiac arrest associated with environmental hypothermia or immersion in icy water can have excellent outcomes despite >30 min of cardiac arrest.[72] One large pediatric study[73] and several smaller studies[74–78] show that good outcome can be achieved when extracorporeal CPR is started after 30–90 min of refractory standard CPR for in-hospital cardiac arrests. The good outcomes were reported primarily in patients with isolated heart disease.

Witnessed events, bystander CPR and a short interval from collapse to arrival of EMS system personnel are important prognostic factors associated with improved outcome in adult resuscitation, and it seems reasonable to extrapolate these factors to children. Children with pre-hospital cardiac arrest caused by blunt trauma[74] and in-hospital cardiac arrest caused by septic shock rarely survive.[78] At least one pediatric study showed that the interval from collapse to initiation of CPR is a significant prognostic factor.[79]

The rescuer should consider whether to discontinue resuscitative efforts after 15–20 min of CPR. Relevant considerations include the cause of the arrest, pre-existing conditions, whether the arrest was witnessed, duration of untreated cardiac arrest (no flow), effectiveness and duration of CPR (low flow), prompt availability of extracorporeal life support for a reversible disease process and associated special circumstances (e.g., icy water drowning, toxic drug exposure).

## CONCLUSION

National and international pediatric resuscitation guidelines are intended to address the unique needs of newborn, infants and children; however, most recommendation are based on expert consensus and extrapolation from adults or (as in the case of hypothermia) neonatal data or studies of pediatric animal models. Historically, pediatric resuscitation focused on prevention of arrest through early recognition and treatment of respiratory failure and shock. Once cardiac arrest occurs, the recommended sequence and emphasis for bystanders and healthcare provider resuscitation is predicated on the prominence of asphysial arrest in the pediatric population, with emphasis on provision of effective ventilation plus effective chest compressions. Although the 2005 international consensus on CPR and ECC science emphasized that rescuers should provide effective chest compression for victims of all ages, there are insufficient pediatric data to evaluate the relative benefits of compression versus ventilations. The information provided in the 2008 AHA science advisory regarding hands-only CPR for adult victims of cardiac arrest raises important questions about the relative benefits of simplified versus tailored sequences for bystander CPR. As we are now in 2010, new guidelines are about to be decided and prospective clinical trials are needed to refine pediatric BLS and ALS resuscitation guidelines to prevent cardiac arrest, define optimal therapies when it does occur and improve survival.
